# The Restauration and Repair of Wounds

**Published:** 1918-08

**Authors:** 


**The Restoration and Repair of Wounds, Combating Contamination and Infection.** Lt-Colonel GEORGE W. CRILE, M. R. C., U. S. A., *Surgery, Gynecology, and Obstetrics*, April, 1918.

The depressed resistance of a contused wound may be most quickly raised by immediate excision of its partially devitalized tissue.

Next in importance, is the application of the great physiologic principle : physiologic rest. It includes more than mere muscular and psychic rest; it implies equally cellular rest. To secure physiologic rest in the case of a fracture, an even, adequate, continous extension must be made. Physiologic rest of the soft parts means quiet produced by means of support. For open wounds it means that antiseptics must not be damaging. It implies no transport, no painful dressings, no alarms.

In the destruction of contaminating bacteria, the first and most dependable agency is the bactericidal power of tissues. This normal defence of tissue against bacteria is present only in living tissues; and the ability of living tissue to overcome infection depends on its vitality. If the defense line is broken at one point, the entire line may give way.

As for antiseptics : the best results are the sum of good surgery and the good use of good chemical antiseptics.

If it is a military necessity th[illegible] patient be rushed down the lines of communication and if the attention of the surgeon is focused away from the principles of surgery and only toward the antiseptic — to the degree that good surgery is out of reach of both the patient and the surgeon — to that extent must antiseptics be depended upon.

An open, fairly superficial wound without inaccessible areas does admirably with normal saline, Carrel-Dakin, " b. i. p. ", eusol or electric light — perhaps best of all by the last named. A wound with deep injury areas will do well treated by the Carrel-Dakin method or " b. i. p. ". In a great rush " b. i. p. " is indicated.

*In the stage of infection*, the treatment consists chiefly in physiologic rest in the broadest sense; and, in addition, if there is sufficient pain, redness and swelling to indicate the actual presence of an invading infection, many and free incisions should be made into the area of infection — throughout the area of infection — until redness and the damaging tension disappears.

If conditions permit, the best single treatment undoubtedly is hot packs; in time of stress " b. i. p. "; in deep wounds, dependent drainage; in quiet times, Carrel-Dakin. When the wounded come in waves, and surgeons and nurses are swamped, incision and " b. i. p. " give the best results to the greatest number per surgeon. But " b. i. p. " must be spread thinly, not applied in masses and the wound should not be sutured but should be lightly packed.

In the granulation and healing stage we have to deal with : (a) contaminated wounds that have become relatively sterile and may be closed; (b) infected wounds that have become relatively sterile and may be closed; (c) wounds with too much loss of tissue ; (d) wounds too deep and too extensive for closure.

The closed wounds require no further discussion. The deep wounds, such as compound fractures, present one fundamental problem, viz. the prevention of the pocketing of pus.

The ideal method by which to secure superficial healing is by immediately covering the surface by skin graft; otherwise, hot packs of eusol or normal saline, alternating with electric light, or sunlight give the best results.

*Meaning of physiologic rest.* Physiologic rest implies no irritating dressings, comfortable position, no compressing bandages, no painful handling, even and balanced muscular pull, no accumulation of wound discharge, apparatus that will permit necessary moving about in bed without breaking physiologic rest.

